# Common Variants at 9q22.33, 14q13.3, and *ATM* Loci, and Risk of Differentiated Thyroid Cancer in the French Polynesian Population

**DOI:** 10.1371/journal.pone.0123700

**Published:** 2015-04-07

**Authors:** Stéphane Maillard, Francesca Damiola, Enora Clero, Maroulio Pertesi, Nivonirina Robinot, Frédérique Rachédi, Jean-Louis Boissin, Joseph Sebbag, Larrys Shan, Frédérique Bost-Bezeaud, Patrick Petitdidier, Françoise Doyon, Constance Xhaard, Carole Rubino, Hélène Blanché, Vladimir Drozdovitch, Fabienne Lesueur, Florent de Vathaire

**Affiliations:** 1 Inserm, Centre for research in Epidemiology and Population Health (CESP), U1018, Radiation Epidemiology Group, F-94800, Villejuif, France; 2 University Paris-Sud, UMRS 1018, F-94807, Villejuif, France; 3 IGR, F-94800, Villejuif, France; 4 Genetic Cancer Susceptibility, International Agency for Research on Cancer (IARC), F-69372, Lyon, France; 5 CRCL, CNRS UMR5286, INSERM U1052, Centre Leon Bérard, Lyon, France; 6 Territorial Hospital Taaone, Papeete, French Polynesia; 7 10 IPRAME, Papeete, French Polynesia; 8 Paofai Clinic, Papeete, French Polynesia; 9 Endocrinologist, Papeete, French Polynesia; 10 Laboratoire Boz, Papeete, French Polynesia; 11 Fondation Jean Dausset CEPH, F-75010, Paris, France; 12 Division of Cancer Epidemiology and Genetics, National Cancer Institute, NIH, DHHS, Bethesda, MD, United States of America; 13 Inserm, U900, Institut Curie, Mines ParisTech, F-75248, Paris, France; Nanjing Medical University, CHINA

## Abstract

**Background:**

French Polynesia has one of the highest incidence rates of thyroid cancer worldwide. Relationships with the atmospheric nuclear weapons tests and other environmental, biological, or behavioral factors have already been reported, but genetic susceptibility has yet to be investigated. We assessed the contribution of polymorphisms at the 9q22.33 and 14q13.3 loci identified by GWAS, and within the DNA repair gene *ATM*, to the risk of differentiated thyroid cancer (DTC) in 177 cases and 275 matched controls from the native population.

**Principal Findings:**

For the GWAS SNP rs965513 near *FOXE1*, an association was found between genotypes G/A and A/A, and risk of DTC. A multiplicative effect of allele A was even noted. An excess risk was also observed in individuals carrying two long alleles of the poly-alanine tract expansion in *FOXE1*, while no association was observed with rs1867277 falling in the promoter region of the gene. In contrast, the GWAS SNP rs944289 (*NKX2-1*) did not show any significant association. Although the missense substitution D1853N (rs1801516) in *ATM* was rare in the population, carriers of the minor allele (A) also showed an excess risk. The relationships between these five polymorphisms and the risk of DTC were not contingent on the body surface area, body mass index, ethnicity or dietary iodine intake. However, an interaction was evidenced between the thyroid radiation dose and rs944289.

**Significance:**

A clear link could not be established between the high incidence in French Polynesia and the studied polymorphisms, involved in susceptibility to DTC in other populations. Important variation in allele frequencies was observed in the Polynesian population as compared to the European populations. For *FOXE1* rs965513, the direction of association and the effect size was similar to that observed in other populations, whereas for *ATM* rs1801516, the minor allele was associated to an increased risk in the Polynesian population and with a decreased risk in the European population.

## Introduction

The incidence of differentiated thyroid cancer (DTC) in French Polynesia, and particularly among women, is one of the highest in the world (37.4 per 100000) [1].

It has been hypothesized that repeated exposure to ionizing radiation may play an important role in the particularly high incidence of DTC in this area. Indeed, a total of 41 atmospheric nuclear weapons tests were carried out above the Mururoa and Fangataufa atolls, between 1966 and 1974Martin 2007. An increased risk of DTC, predominantly of the papillary type, was already observed among the survivors of the two bombings of Hiroshima and Nagasaki in 1945 [2–5], among persons exposed after the Chernobyl accident [6–9], and also in patients treated with radiotherapy [10–12]. To evaluate the impact of the nuclear weapons tests fallout, we recently conducted a large population-based case-control study in French Polynesia. The external and internal radiation exposure was evaluated for 602 native Polynesians, all under the age of 15 in 1974 [13]. The average dose received to the thyroid gland was very low, but the DTC risk was shown to slightly increase with it [14].

However, nuclear fallouts could explain only a small part of the high incidence. Other risk factors such as a family history of thyroid cancer, menstrual and reproductive factors, weight and body mass index (BMI), body surface area (BSA), dietary iodine intake or dietary consumption have also been investigated in the same study and have shown to modify the risk of DTC [15–20]. Genetic factors may also contribute to DTC susceptibility, but they have never been assessed in this population.

Both *NKX2-1* (*NK2 homeobox 1*, also called *TTF1* for *Thyroid Transcription Factor 1*) and *FOXE1 (Forkhead factor E1*, also called *TTF2* for *Thyroid Transcription Factor 2)* encode thyroid-specific transcription factors. They play a crucial role in the development of the thyroid gland and their expression is modified in thyroid tumors [21–24]. In 2009, a genome-wide association study (GWAS) reported the contribution of two SNPs nearby *NKX2-1* and *FOXE1* to the risk of developing DTC in Icelandic and European populations [25]. The first SNP, rs944289, is located 337-kb upstream of the *NKX2-1* gene and was described to increase the risk of DTC [25–26]. The second SNP, rs965513, is located in an intergenic region, 57-kb upstream of the *FOXE1* gene. It has been related to many endocrine and metabolic disorders [27], to changes in the concentration of thyroid hormones [25], and to an increased risk of DTC [25–26,28]. Rs965513 has been reported as a strong genetic factor for both sporadic and radio-induced papillary thyroid cancer (PTC) [29]. Located within the 5' untranslated region (UTR) of *FOXE1*, the SNP rs1867277 has been reported as a risk factor for DTC and has been suggested as the causal SNP [28,30]. It has been also proposed that the minor allele A could modulate a transcriptional regulation pathway of the *FOXE1* gene, by recruiting USF1/USF2 transcription factors [31]. Subsequently, association between the poly-alanine expansion within the transcription factor FOXE1 (rs71369530) was reported and transcriptional analyses have also suggested a functional implication of this multi-allelic polymorphism. Indeed, Carré et *al*. observed different activities and different relations to the risk of thyroid disorder according to the number of alanine repeats [31]. This variable length polymorphism was found in tight linkage disequilibrium with rs1867277 and may be responsible for the strong association that is often observed between *FOXE1* and papillary thyroid carcinoma (PTC) [32].

The *ATM* (*Ataxia-Telangiectasia Mutated*) gene is involved in the repair of DNA double-strand breaks. Mutations in this gene have been related to ataxia telangiectasia and other disorders, especially characterized by radiation sensitivity and cancer predisposition [33]. Variations in this gene were reported to play a role in hormone cancers [34–35]. An association between the missense substitution D1853N (rs1801516) and a reduced risk of PTC was also reported for both sporadic and radiation-induced thyroid cancer [36]. Recently, we also showed that this coding SNP in *ATM* contributes to the risk of PTC in Belarusian children exposed to ionizing radiation from the Chernobyl nuclear power plant accident [37].

We thus sought to investigate the contribution of genetic variations at the *NKX2-1*, *FOXE1* and *ATM* loci to the risk of DTC in the Polynesian population, which is geographically isolated and where residents were repetitively exposed to ionizing radiation during the nuclear tests.

## Results

The genotypes distribution conformed to Hardy-Weinberg Equilibrium (HWE) for both cases and controls, for all of the investigated SNPs, except the *NKX2-1* SNP rs944289 (Table 1). The Minor Allele Frequency (MAF) in cases and in controls is also given for each SNP in Table 1.

**Table 1:** Description of the five studied polymorphisms.

					Participants (N = 452)			
			Polymorphism		Minor Allele Frequency		Hardy-Weinberg Equilibrium χ^2^ p-value	
Reference	Location	Chromosome	Allele change	Residue change	Cases	Controls	Cases	Controls
rs944289	Intergenic, 337 kb telomeric of *NKX2-1*	14q13.3	C>T	-	0.32	0.27	0.01	0.001
rs965513	Intergenic, 57 kb upstream to *FOXE1*	9q22.33	G>A	-	0.27	0.21	0.27	0.97
rs1867277	5’UTR of *FOXE1*	9q22.33	G>A	-	0.20	0.19	0.43	0.64
rs71369530	*FOXE1*	9q22.33	Poly-alanine tract expansion		0.17	0.15	0.49	0.47
rs1801516	Missense substitution at codon 1853 of *ATM*	11q22-23	G>A	D[Asp]>N[Asn]	0.03	0.02	0.67	0.80

A 3-fold increased risk of developing DTC was found for subjects carrying the A/A genotype for the GWAS SNP rs965513 near *FOXE1*, compared to the G/G genotype (OR = 3.32, p = 0.02, under co-dominant model) (Table 2). For the *FOXE1* length polymorphism, a 4-fold increased risk of developing DTC was observed for carriers of the L/L genotype, compared to the S/S genotype (OR = 4.16, p = 0.04, under co-dominant model). No association was found with the other proposed functional SNP located in the promoter region of *FOXE1* (rs1867277), unlike reported by others in other populations.

**Table 2:** Association results between the five polymorphisms and the risk of developing DTC.

	Genotyped participants					
Genotypes	Cases n (%)	Controls n (%)	Crude OR ^a^ (95% CI)	p-value	Adjusted OR ^b^ (95% CI)	p-value
rs944289 (near *NKX2-1*)	n = 168	n = 262				
C/C	84 (50)	149 (56.9)	1.00		1.00	
C/T	59 (35.1)	83 (31.7)	1.27 (0.82–1.95)	0.9	1.33 (0.85–2.08)	0.9
T/T	25 (14.9)	30 (11.4)	1.50 (0.82–2.73)	0.3	1.66 (0.88–3.12)	0.2
Risk per T allele ^c^			1.23 (0.94–1.63)	0.1	1.30 (0.97–1.74)	0.08
C/T+T/T *versus* C/C ^d^			1.33 (0.89–1.96)	0.2	1.41 (0.93–2.13)	0.1
T/T *versus* C/T+C/C ^e^			1.36 (0.76–2.41)	0.3	1.48 (0.81–2.70)	0.2
rs965513 (near *FOXE1*)	n = 160	n = 248				
G/G	89 (55.6)	155 (62.5)	1.00		1.00	
G/A	57 (35.6)	82 (33.1)	1.21 (0.79–1.85)	0.4	1.25 (0.80–1.94)	0.2
A/A	14 (8.8)	11 (4.4)	2.33 (1.01–5.35)	0.07	**3.32 (1.34–8.20)**	**0.02**
Risk per A allele ^c^			1.36 (0.99–1.88)	0.06	**1.50 (1.06–2.12)**	**0.02**
G/A+A/A *versus* G/G ^d^			1.33 (0.89–2.00)	0.2	1.43 (0.94–2.17)	0.1
A/A *versus* G/A+G/G ^e^			**2.17 (0.96–4.91)**	**0.06**	**3.04 (1.25–7.38)**	**0.01**
rs1867277 (5’UTR of *FOXE1*)	n = 137	n = 222				
G/G	89 (65.0)	147 (66.2)	1.00		1.00	
G/A	41 (29.9)	66 (29.7)	1.06 (0.66–1.69)	0.7	1.13 (0.68–1.85)	0.7
A/A	7 (5.1)	9 (4.1)	1.39 (0.50–3.85)	0.6	1.72 (0.57–5.17)	0.4
Risk per A allele ^c^			1.11 (0.77–1.61)	0.6	1.20 (0.81–1.79)	0.4
G/A+A/A *versus* G/G ^d^			1.10 (0.70–1.71)	0.7	1.19 (0.74–1.91)	0.5
A/A *versus* G/A+G/G ^e^			1.37 (0.50–3.76)	0.6	1.66 (0.56–4.94)	0.4
rs71369530 (length polymorphism in *FOXE1*)	n = 165	n = 258				
S/S^f^	115 (69.7)	187 (72.5)	1.00		1.00	
S/L^f^	44 (26.7)	67 (26.0)	1.08 (0.69–1.69)	0.3	1.05 (0.66–1.68)	0.1
L/L^f^	6 (3.6)	4 (1.5)	2.56 (0.71–9.20)	0.17	**4.16 (1.07–16.1)**	**0.04**
Risk per L sequence ^c^			1.23 (0.84–1.79)	0.29	1.29 (0.87–1.92)	0.2
S/L+L/L *versus* S/S ^d^ ^.^ ^f^			1.17 (0.76–1.79)	0.48	1.18 (0.76–1.85)	0.46
L/L *versus* S/L+S/S ^e^ ^.^ ^f^			2.51 (0.70–8.99)	0.16	**4.11 (1.07–15.9)**	**0.04**
rs1801516 (*ATM*)	n = 175	n = 270				
G/G	164 (93.7)	262 (97.0)	1.00		1.00	
G/A	11 (6.3)	8 (3.0)	2.22 (0.88–5.6)	0.09	3.13 (1.17–8.31)	0.02

^a^ Stratified by age and sex.

^b^ Stratified by age and sex, and adjusted on BMI, BSA ethnicity, and thyroid radiation dose received before age 15 years.

^c^ Multiplicative model of inheritance.

^d^ Dominant model of inheritance (combined heterozygotes and rare homozygotes *versus* common homozygotes).

^e^ Recessive model of inheritance (rare homozygotes *versus* combined heterozygotes and common homozygotes).

^f^ S for alleles coding for 12–14 alanines and L for alleles coding for 16–19 alanines.

The *ATM* missense substitution D1853N (rs1801516) was much rarer in the Polynesian population than in the European populations (MAF = 0.02). Nonetheless, an excess risk of DTC was detected for carriers of the minor allele (A) (OR = 3.13, p = 0.02).

Finally, no association was found in the Polynesian population for the GWAS SNP rs944289 near *NKX2-1*.

Compared to controls, the DTC cases had a higher BMI (OR = 2.50, 95%CI = 1.66–3.75, p<0.001) and a higher BSA (OR = 2.70, 95%CI = 1.78–4.08, p<0.001). With regard to exposition to nuclear tests, cases did not receive thyroid radiation dose higher than 2.0 mGy before the age of 15 more frequently than controls (OR = 1.24, 95%CI = 0.71–2.17, p = 0.4). The relationship between the five tested polymorphisms and the risk of DTC was not contingent on BMI, BSA, ethnicity or dietary iodine intake (Table 3). Despite the small number of investigated subjects, a significant interaction was evidenced between the thyroid radiation dose and *NKX2-1* rs944289 when considering the T/T genotype (p = 0.04). Hence, among subjects who received 2 mGy or less to the thyroid gland during the nuclear tests, the risk of DTC was similar in subjects with the T/T genotype to others (OR = 0.99, 95%CI 0.52–1.88), whereas among subjects who received more than 2 mGy, subjects with the T/T genotype had a much higher risk than others (OR = 6.13, 95%CI 1.21–31.3). Similarly, a thyroid radiation dose > 2mGy had no significant effect on subjects with C/C or C/T genotype (OR = 1.12, 95%CI 0.63–2.00) but was associated with an increased risk in the T/T homozygous subjects (OR = 6.94, 95%CI 1.31–37.0).

**Table 3:** Results of interaction tests between genetic factors and other putative risk factors for DTC.

		rs944289 (near *NKX2-1*)			p-interaction	rs965513 (near *FOXE1*)			p-interaction	rs1867277 (5’UTR of *FOXE1*)			p-interaction	rs71369530 (microsatellite in *FOXE1*)*			p-interaction	rs1801516 (*ATM*)		p-interaction
		C/C	C/T	T/T		G/G	G/A	A/A		G/G	G/A	A/A		S/S	L/S	L/L		G/G	G/A	
Ethnicity					0.8				0.9				0.7				0.3			1.0
Participants with Polynesian parents	Cases	72	50	20		72	51	12		75	35	5		98	39	4		139	10	
	Controls	132	64	26		127	71	11		121	55	8		156	60	4		221	8	
Participants with parents of mixed origin	Cases	12	9	5		17	6	2		14	6	2		17	5	2		25	1	
	Controls	17	19	4		28	11	0		26	11	1		31	7	0		41	0	
Body Mass Index (kg/m^2^)					0.4				0.9				0.1				0.9			0.2
> Median in genotyped controls	Cases	63	42	14		64	42	7		69	30	2		83	33	2		116	8	
	Controls	78	39	14		79	39	3		76	29	5		94	34	0		133	2	
≤ Median in genotyped controls	Cases	21	17	11		25	15	7		20	11	5		32	11	4		48	3	
	Controls	71	44	16		76	43	8		71	37	4		93	33	4		129	6	
Body Surface Area (m^2^)					0.3				0.8				0.3				0.5			0.2
> Median in genotyped controls	Cases	64	44	13		65	42	9		67	33	2		84	34	2		119	7	
	Controls	82	33	16		75	43	4		75	26	6		92	35	1		135	1	
≤ Median in genotyped controls	Cases	20	15	12		24	15	5		22	8	5		31	10	4		45	4	
	Controls	67	50	14		80	39	7		72	40	3		95	32	3		127	7	
Dietary Iodine Intake (μg/day)					0.3				0.8				0.6				0.6			0.3
> Median in genotyped controls	Cases	37	28	10		37	25	6		43	14	4		55	17	3		73	5	
	Controls	80	41	10		79	39	6		79	25	8		96	29	4		126	6	
≤ Median in genotyped controls	Cases	47	31	15		52	32	8		46	27	3		60	27	3		91	6	
	Controls	69	42	20		76	43	5		68	41	1		91	38	0		136	2	
Thyroid radiation dose received before age 15 years (mGy)					0.1				0.9				0.9				0.9			0.9
> 2 mGy	Cases	17	9	8		17	9	4		18	9	1		23	8	1		34	0	
	Controls	32	9	2		28	11	3		23	11	1		34	9	1		43	3	
≤ 2 mGy	Cases	67	50	17		72	48	10		71	32	6		91	36	5		130	11	
	Controls	117	74	28		127	71	8		124	55	8		149	57	3		219	5	

* S for Short alleles (12–14 alanines) and L for Long allele (16–19 alanines).

## Discussion

In the present work, we assessed the relationship between five putative or recognised genetic susceptibility markers for DTC in French Polynesia, where the thyroid cancer incidence is among the highest worldwide [1]. To our knowledge, this was the first time that such a study on genetic susceptibility to cancer was undertaken in the population of this French overseas territory, which is unique notably because of a repeated exposure to nuclear weapons tests and a relative geographical isolation.

Because of its very low MAF in controls (2%), the power of our study for evidencing an association between *ATM* SNP rs1801516, and DTC could reach 80% only for an OR of 3.5 or higher. For the other tested SNPs, MAF in controls was about 20% (range 15% to 27%, Table 1), and our study had a power of 80% for evidencing an association if OR is about 1.7. Hence, our study had a sufficient power (80%) for evidencing gene-environment interactions only for a factor of about 3 or higher, in the best situation (environmental factor frequency = 50%, main OR for environmental factor = 2, MAF = 20%, main OR per minor allele = 1.5).

The association we observed between *FOXE1* or *ATM* and DTC risk in the Polynesian population is not surprising given that we focused on polymorphisms that had been previously found to be associated with DTC in others populations in several studies. Nevertheless, we cannot exclude the possibility of false positive results. Indeed, the p-values of our positive results ranged from 0.01 to 0.04, and did not remain significant when correcting for the multiple (n = 5) tests using Bonferoni correction. Unfortunately, our results could not be compared to others obtained in genetically similar populations, because to our knowledge the SNPs we investigated had not been so far addressed in DTC in other pacific islands populations.

Interestingly, we did not replicate the association with the GWAS SNP rs944289 at the *NKX2-1* locus on chromosome 14q13.3, as reported by others in Icelandic and European populations, but a significant interaction was observed between the thyroid radiation dose before the age of 15 and the homozygotes for this polymorphism (p = 0.04). However, it should be noted that the distribution of the rs944289 genotypes deviated from HWE in both case and control groups. Therefore, this result on interaction should be interpreted with caution. Deviations from HWE may point to either a sampling bias, mistyping of genotypes, or spurious gene associations because of population stratification. We ruled out a technical genotyping issue because the same HRM probe and protocol were used in other studies on different populations and HWE proportions were respected in them ([37], Lesueur *et al*., unpublished data). Moreover, we have re-sequenced a subset of the Polynesian samples to confirm genotypes. Another possible explanation could be a bias due to the poor quality of some DNA samples. Indeed, in our study, 68% of the DNA samples were prepared from buccal swab brushes and 32% from Oragene kits. DNA samples prepared from Oragene kits are usually less degraded than those prepared from buccal swab brushes. We thus re-analysed the SNP rs944289 considering only individuals with DNA samples prepared from Oragene kits. In this subgroup composed of 145 individuals, the genotype distribution conformed to HWE.

We could also hypothesize that deviation from HWE comes from the existence of sub-groups in the Polynesian population, with high rates of homogamy and consanguinity. Indeed, French Polynesia comprises 118 main islands, of which 76 are inhabited. Despite the recent improvement of airlines, French Polynesians remain relatively isolated from each other and a particular structuring of the population could not be excluded, with putative genetic consequences. However, this deviance was not observed with the others four investigated polymorphisms.

Among all of the successfully genotyped participants of the study, we observed MAFs equal to 0.32 for cases and 0.27 for controls for the SNP rs944289. Compared to the frequency reported in the NCBI dbSNP database (MAF_NCBI_ = 0.43) and to those reported in other studies [25–26], the frequency in Polynesian individuals was clearly lower.

The thyroid specific transcription factor FOXE1 plays a major role in the morphogenesis of the thyroid gland and in the regulation of thyroid differentiated state maintenance [22,38]. DNA sequence variations within the *FOXE1* gene have been repeatedly associated with susceptibility to DTC but these variations have been so far mostly studied in Icelandic, European and Japanese populations. In the Polynesian population, the MAF of the GWAS SNP rs965513 (0.21) was similar in controls to the observed MAF in European populations [27], that is intermediate to the MAF observed in the Icelandic population (MAF = 0.34) [26] and the MAF observed in the Japanese population (MAF = 0.057) [27]. In French Polynesia, the A/A genotype was also associated with a significant excess risk of developing DTC and the size effect of this SNP was similar to what was observed elsewhere, confirming that rs965513 represents a robust susceptibility marker for DTC.

The length polymorphism rs71369530 is caused by an expansion of a poly-alanine stretch in the FOXE1 protein with consequences on its activity [31–32]. Although the frequency of short alleles (S) in cases was similar to the frequency found in French cases by Carré *et al*. (0.17 vs. 0.20), the MAF in controls was almost the half that of the MAF found in the French controls of the same study (0.15 vs. 0.35) [31]. In the Polynesian population, we found a highly increased DTC risk associated with the L/L genotype. No significant interaction with other DTC risk factors was established here. As the intergenic GWAS SNP rs965513, the length polymorphism rs71369530 within *FOXE1* represents a susceptibility marker for DTC and reinforces the hypothesis that *FOXE1* is the DTC susceptibility gene at locus 9q22.33.

The SNP rs1867277 in the 5’UTR of *FOXE1* has been also proposed as the causal SNP involved in the susceptibility to DTC, because it has been related to a modulation of the transcriptional regulation of the gene [28,30]. Interestingly in our study, no association with DTC was found. This result is quite important because it highlights the value of conducting association studies in different populations when performing fine-mapping to identify causal variants. Indeed, in the former published studies, rs1867277 and rs71369530 appeared to be strongly correlated and it was difficult to disentangle the involvement of the respective polymorphisms. In the Polynesian population, the two polymorphisms were clearly not in strong linkage disequilibrium. In French Polynesia, the MAF of rs1867277 was specific of the Polynesian population (0.19 in controls versus 0.39 in Europeans [28], and 0.40 in Spanish controls [30]).

As for *NKX2-1* rs944289 and *FOXE1* rs1867277, the MAF of *ATM D1853N* (rs1801516) differed quite a lot from MAF observed in other populations (MAF_Caucasians_ = 0.19 and MAF_Polynesians_ = 0.02). Notwithstanding, the minor allele (A) was significantly associated with an excess risk of DTC among Polynesians subjects (OR = 3.13, 95%CI = 1.17–8.31). This result was intriguing since a moderate decline in the risk associated with the A allele (OR = 0.69, 95%CI = 0.45–0.86, p-value = 0.03 in 255 Chernobyl radiation-induced or sporadic PTC cases and 596 controls, all from Caucasian origin [36], as well as in a case-control study sampled from children living in the area contaminated by fallout from the Chernobyl power plant accident [37]. The effect of rs1801516 and its role in the modulation of the ionizing radiations effect have long been debated and the results are still conflicting. An association of the minor allele (A) was found with radio-sensitivity in breast cancer patients [39] and this result was supported by a study showing an increased radio-sensitivity of human fibroblasts carrying allele A [40]. By contrast, a protective role of allele A was found on the adverse side effects of radiotherapy [41].

In conclusion, we confirmed the contribution of *FOXE1* and *ATM* genes in the etiology of DTC in French Polynesia. The relevance of the intergenic SNP rs965513 on chromosome 9q22.33 and the poly-alanine tract polymorphism rs71369530 in *FOXE1* as robust susceptibility markers for DTC was evidenced in the present study. The role of the missense substitution D1853N in the DNA repair gene *ATM* in population exposed to radiation is still under debate, since results in the Polynesian population are not consistent with the previously described associations between the *ATM* coding SNP and a reduced risk of DTC.

Although the studied Polynesian population showed some genetic particularities, in terms of allele frequency, association or interaction, we did not found any major genetic factor likely to explain the high incidence rate observed in the territory. Previous studies in this population reported the important role of anthropomorphic, environmental and behavioral factors as risk factors for DTC. All of these results suggest a cumulative effect. Moreover, we hypothesize that some of these risk factors may interact, in the manner observed between the thyroid radiation dose and *ATM* missense substitution D1853N. Finally one cannot exclude that other sequence DNA variation such as epigenetic changes may occur after exposure to some environmental factors such as ionizing radiation that could lead to the development of DTC.

## Subjects, Material and Methods

### Study population

The study was conducted on a sub-group of subjects from a population-based case-control study carried out in French Polynesia to assess risk factors of DTC potentially involved in this population [18].

Written informed consent was obtained from all participants. The study was carried out with the written agreement of the Ethical committee of French Polynesia and the French “Commission Nationale de l’Informatique et des Libertés” (CNIL), which had previously approved the contact procedure and the consent form, and included the possibility for contacting their medical doctor. All participants were major (> = 18y) at time of interview. All documents, including consent forms and questionnaires, are conserved in a secured cabinet in the premises of the 1018-INSERM Unit U1018.

Six hundred and two subjects participate in the initial case-control study (characteristics of participants were detailed in Clero *et al*, 2012 [18] and summarized in Table 4). All subjects were born in and residents of French Polynesia. Cases were diagnosed for thyroid carcinoma between 1979 and 2004. Among them, 177 presented the histology of papillary thyroid carcinoma and 52 the histology of follicular thyroid carcinoma. Controls were healthy individuals, selected from the native population and matched with cases, according the date of birth and gender. The selection of controls was directly performed from the registry of births, covering the whole country. The completeness was possible because of the small size of the local population (264 736 inhabitants in 2007). It allowed us to contact almost all potential controls, even simply with the name, date or place of residence. Five hundred and twenty participants had two parents of Polynesian origin and 82 of them had one Polynesian parent and one non-Polynesian parent (*i*.*e*. of Asiatic, European or other ethnic origin).

**Table 4:** Characteristics of the 602 participants of the case-control study, born and resident in French Polynesia, and of the sub-group included in the genetic study.

	All (N = 602)		Genotyped participants (N = 452)	
Characteristics	Controls N (%)	Cases N (%)	Controls N (%)	Cases N (%)
Gender				
Male	47 (12.6)	26 (11.4)	31 (11.3)	21 (11.9)
Female	326 (87.4)	203 (88.6)	244 (88.7)	156 (88.1)
Age at diagnosis (years)				
<25	38 (10.2)	23 (10.0)	28 (10.2)	20 (11.3)
25–29	42 (11.3)	24 (10.5)	29 (10.6)	17 (09.6)
30–34	46 (12.2)	30 (13.1)	36 (13.1)	22 (12.4)
35–39	76 (20.4)	46 (20.1)	58 (21.1)	36 (20.3)
40–44	54 (14.5)	32 (14.0)	39 (14.2)	26 (14.7)
45–49	53 (14.2)	31 (13.5)	38 (13.8)	22 (12.4)
≥ 50	64 (17.2)	43 (18.8)	47 (17.0)	34 (19.3)
Histology				
Papillary thyroid carcinoma		177 (77.3)		135 (76.3)
Follicular thyroid carcinoma		52 (22.7)		42 (23.7)
Ethnicity				
Participants with Polynesian parents	320 (85.8)	200 (87.3)	234 (85.1)	151 (85.3)
Participants with parents of mixed origin	53 (14.2)	29 (12.7)	41 (14.9)	26 (14.7)
Body Mass Index (kg/m^2^)				
> Median in genotyped controls	193 (51.7)	161 (70.3)	137 (49.8)	125 (70.6)
≤ Median in genotyped controls	179 (48.0)	68 (29.7)	138 (50.2)	52 (29.4)
Missing data	1 (0.3)	0	0	0
Body Surface Area (m^2^)				
> Median in genotyped controls	194 (52.0)	162 (70.7)	138 (50.2)	127 (71.8)
≤ Median in genotyped controls	178 (47.7)	67 (29.3)	137 (49.8)	50 (28.2)
Missing data	1 (0.3)	0	0	0
Dietary iodine intake (μg/day)				
> Median in genotyped controls	188 (50.4)	101 (44.1)	136 (49.5)	78 (44.1)
≤ Median in genotyped controls	185 (49.6)	128 (55.9)	139 (50.5)	99 (55.9)
Thyroid radiation dose received before age 15 years (mGy)				
> 2	70 (18.8)	47 (20.5)	47 (17.1)	34 (19.2)
≤ 2	303 (81.2)	182 (79.5)	228 (82.9)	143 (80.8)

All study participants were interviewed. Epidemiological and anthropological information were collected, including height, weight, ethnic origin of parents, and dietary habits. A detailed estimation of radiation doses received at different ages during atmospheric nuclear tests has been performed [13], based on the radioactive fallout data published by France at the end of each year of tests, on ^131^I and ^137^Cs counts in fresh milk and of total g-activity and ^137^Cs in vegetables and fish, and on meteorological data 21 days before and seven days after each test. Estimation of the water level in cisterns before each test, size of the cisterns and information on wind and rain were also used. For each study participant, the total thyroid dose was the sum of the thyroid doses reconstructed for each test, taking into account age, source of drinking water and data collected in the diet questionnaire. The individual consumption of each foodstuff at a given age was deduced from the information on consumption during childhood obtained in the interview, using age-specific scaling factors. Dose reconstruction was conducted without knowledge of the case or control status of the subject.

Four hundred and fifty two participants (177 cases and 275 controls) gave their consent for biological sampling and genetic analyses. For 307 of them, buccal cells were sampled using Epicentre Biotechnologies MasterAmp buccal swab brushes (Madison, WI, USA), and for the 145 others, a saliva sample was collected using a DNA Genotek Oragene DNA collection kit (Ottawa, Canada). Genomic DNA (gDNA) was extracted from these samples with a Qiagen Autopure LS (Courtaboeuf. France). The gDNA was then quantified with Life Technologies Picogreen (Saint-Aubin, France).

### Genotyping

For SNP rs965516 and rs1867277, 10 ng gDNA were analysed using a specific TaqMan Pre-designed SNP Genotyping Assay (Applied Biosystems, Foster City, CA, USA). Fluorescence readings and data analyses were performed with the ABI PRISM 7900HT Sequence detection system. For rs944289 and rs1801516, 25ng gDNA were analysed using High-Resolution Melting curve (HRM), with a specific probe. Some representative samples were re-sequenced by dye-terminator to confirm the genotype [42]. Fluorescence readings and data analyses were done with the Idaho Technology LightScanner Inc. Hi-Res Melting System.

The length polymorphism rs71369530 in *FOXE1* is due to a variable number of alanine repeats. Thirty nanograms of gDNA was amplified by PCR with fluorescently end-labelled forward primers (5'-6-FAM or 5'-HEX), by using KAPA 2G Fast HotStart ReadyMix (KAPA Biosystems, Woburn, MA, US) in a 10 μl-final reaction volume (0.5 mM MgCl_2_, 5%DMSO, 0.25 μM primers). The fluorescently-labelled PCR product was loaded on an ABI 3730 capillary sequencer and analysed as a variable length fragment polymorphism using GenScan size standard ROX-500 as internal size standards. Data were collected and visualised with Genotyper Software v3.7. To determine the number of repeats that corresponds to each allele identified in the genotyping assay, the PCR products from 6 homozygous individuals were Sanger sequenced.

The sequences of all PCR primers, HRM probes, TaqMan probes, and all PCR conditions are available from the authors on request.

The proportion of successfully genotyped DNA samples was 95.1% for rs944289 (near *NKX2-1*), 90.3% for rs965513 (near *FOXE1*), 79.4% for rs1867277 (5’UTR of *FOXE1*), 93.6% for rs71369530 (poly-alanine expansion in *FOXE1*) and 98.5% for rs1801516 (*ATM*), respectively. Raw genotyping data are available in S1 Table.

### Statistical analyses

Although this case-control study was matched one, each case being matched to 1 to 2 controls on age and sex, we were not able to use conditional regression analysis because DNA was not collected for all cases and controls and this would conduct to a eliminate several strata because of DNA missing either for the case or for each of the controls. The association between these five polymorphisms and the risk of DTC was assessed using multiple logistic regressions, stratified by age and gender, and assuming co-dominant, dominant, and recessive genetic models of inheritance [43–44]. Crude analyses and analyses adjusted for BMI, BSA, ethnicity, and radiation dose were conducted. Tests for interaction were performed to determine whether the putative associations of SNPs with the risk of developing DTC were modified by parameters such as BSA, BMI, ethnicity, radiation dose, and dietary iodine intake [43]. All statistical analyses were done with the SAS software, version 9.3 (SAS Institute Inc, NC, USA).

Age distribution of study participants, at time of thyroid cancer diagnosis or corresponding age for controls, was ranged from 10 to 62 and was divided into seven homogeneous classes, which were then used along with gender to stratify the cases and controls for statistical analyses. Two groups were designed for ethnic origin: those individuals with two Polynesian parents and others. For BMI, BSA, and dietary iodine intake, the medians of the distributions among the female genotyped controls (25.7 kg/m^2^, 1.8 m^2^, 132.2 μg/day, respectively) and the medians of the distributions among the male genotyped controls (30.1 kg/m^2^, 2.1 m^2^, 146.9 μg/day, respectively) were used as limits to create two groups among the participants in our study. Finally, the individuals were also grouped into two sets for the radiation dose received to the thyroid before the age of 15, according to whether the dose was under or above the median dose estimated among the genotyped controls (0.37 mGy).

Genotype frequencies and Minor Allele Frequencies (MAFs) were calculated in cases and in controls. They were then evaluated for departure from Hardy-Weinberg Equilibrium using a χ^2^ test (see Table 1). The G/G genotype for rs965513, rs1867277, and rs1801516, and the C/C genotype for rs944289 were considered ancestral, because they were the most frequent genotypes in the Polynesian population. For the *FOXE1* poly-alanine stretch polymorphism, the different allelic sizes were encoded in two categories: short and long alleles. The short alleles (S) included the alleles coding for a stretch of 12–14 alanines, while the long alleles (L) comprised those alleles coding for a stretch of 16–19 alanines (Table 5). Because the short alleles were more common in the studied population, the S/S genotype was the pattern selected to be the reference.

**Table 5:** Observed frequencies of FOXE1 multi-allelic poly-alanine tract alleles (rs71369530) in the Polynesian population.

Genotype	Genotype (recoded)	Cases n = 165	Controls N = 258
12 Ala / 16 Ala	S / L	1	1
14 Ala / 14 Ala	S / S	115	187
14 Ala / 16 Ala	S / L	42	66
14 Ala / 17 Ala	S / L	1	0
16 Ala / 16 Ala	L / L	6	4

## Supporting Information

S1 Table Genotyping data obtained for the 5 tested polymorphisms. (XLSX)
